# Some Diseases of the Eye in Lower Animals

**Published:** 1883-01

**Authors:** William Oliver Moore

**Affiliations:** Prof. Comparative Ophthalmology, Columbia Veterinary College, N. Y., Assistant Surgeon New York Eye and Ear Infirmary, etc.; 133 East 38th Street, N. Y. City


### Art. V.—SOME DISEASES OF THE EYE IN LOWER ANIMALS.

BY WILLIAM OLIVER MOORE, M.D.,
*Prof. Comparative Ophthalmology, Columbia Veterinary College, N. Y., Assistant Surgeon New York Eye and Ear Infirmary, etc.*

IT is only recently that the diseases of the eye of the lower animals have engaged the attention of the specialists, who devote themselves to the study of the causes, nature and cure of the numerous affections to which the eye is subject. With the exception of a few special diseases the inferior animals have most of the ophthalmic affections common to man. No doubt the list of affections of the eye of horses, cattle, sheep and swine might be extended if we would more carefully employ the improved means of observation, so constantly used by the ophthalmologist. The horse whose eyes are a special subject of solicitude, is credited with more diseases of this organ than all the rest of the animals on the farm put together. The ophthalmoscope is one of the most valuable aids in determining diseases of the interior of the eye, and those few veterinarians who have learned to use it entertain no doubt of its great value. The instrument has not, however, come yet into general use, but soon will, as each year the students of Columbia Veterinary School are instructed on the living subject. In examining a horse for soundness the ophthalmoscope should be used, for, latent disease of the interior of the eye can only thus be determined; by it, it has already been shown that Periodic Ophthalmia in the horse is not an interrupted, but a continuous disease, viz: a choroiditis. Another method of examining the eye is by a two inch convex lens, which is used to condense the light and then illuminate the external parts of the organ—sunlight or artificial light may be employed—it is called oblique illumination. It is especially serviceable in detecting foreign substances, hayseed and the like, on the cornea or conjunctiva. By the aid of these two means, the ophthalmoscope and the oblique illumination, we can detect any disease, either of the internal or external parts of the eye. Having thus these means of observation and aids to diagnosis, let us look at some of the more common affections of this organ.

*Simple Conjunctivitis*—In other words an inflammation of the mucous membrane, lining the eye-lids and covering in part the eye-ball; a membrane continuous with the pharyngeal mucous membrane through the nasal duct. Thus many cases of conjunctivitis arise from common cold and influenza by continuity of tissue.

It is a very common form of disease in all animals, and although simple in its nature, it gives rise to a great deal of temporary inconvenience owing to the extreme sensibility of the conjunctiva.

It most commonly is caused by cold, but may be induced by various means, as a slight blow, the contact of a twig when the horse is in motion; particles of dust may be accidentally lodged on the eye, exposure to the sun's rays, especially if the soil is bare of pasture, all of which deserve recognition.

The disease is attended with well marked symptoms. First there is intolerance of light (photophobia), with marked injection of the palpebral and ocular conjunctiva, increased flow of tears, with some swelling of the eye-lids, which have an increased temperature. On everting the lids, especially the upper, the mucous membrane is found to be congested and the retro-tarsal fold swollen; in some cases the ocular conjunctiva is swollen and presents the appearance of a "water blister," this condition is known as chemosis.

Mucous secretion in increased quantity soon makes its appearance, the amount varying with the severity of the disease.

There is generally no pain, but rather the sensation of heat and itching, the eye feeling as if particles of sand were under the lid. As a rule, in simple cases the cornea is not at all effected, but where the œdema of the ocular conjunctiva is great it may become milky in appearance, owing to the malnutrition of the part, this generally disappears on the subsidence of the inflammation. In animals this milkness of the cornea clears up remarkably quick, whereas in the human subject it is very slow to yield. The vision is generally interfered with owing to the fear of light, and in the horse the thrusting forward of the nictitating membrane. In some cases the secretion agglutinates the lids together, and in others only collects in the inner canthus.

Conjunctivitis from cold usually occurs in both eyes at once, if from injury in one or both, according to the nature of the case. The only disease likely to be mistaken for it is periodic ophthalmia (iridochoroiditis) in its onset; if we remember, however, that the latter occurs in one eye only, as a rule, at first, we will avoid mistakes.

The treatment of simple conjunctivitis consists in most cases of local measures alone. Rest is very essential if the case is very marked, and with it pure air is an adjunct. A horse confined in an ill-ventilated stable is in a fair way to have his conjuntivitis kept up from the ammoniacal vapor.

Various astringents may be used; a favorite one being a solution of one drachm of pulverized alum to one pint of water, dropping in three or four drops upon the eye-lid every three hours.

Where this does not act well, nitrate of silver, five or ten grains to water one ounce, may be used by means of a camel's hair pencil, everting the upper lid and then carefully applying it. The application of water, ice cold, by means of a sponge or cloth to the closed eye-lids adds very much to the comfort of the patient. These may be used every two hours, continuing them from ten to fifteen minutes.

Generally with the use of the cold water and alum solution most mild cases subside in a few days. Should they not the silver solution may be substituted.

*Purulent Conjunctivitis.*—Some inflammations starting as catarrhal degenerate and become purulent in character, having an increase in all the symptoms.

A purulent inflammation of the conjunctiva occurs in some seasons of the year among cattle and sheep which are feeding in low, damp situations, especially in spring and autumn during the prevalence of rain with cold winds.

When the affection assumes an epizootic form it is termed "blinds."

The existence of "blinds" among animals at a time when no special cause could be detected, led to the theory that it was due to a variety of gadfly, which it is supposed deposits its ovum in the centre of the cornea and thus causes the irritation.

Some ground for this theory is furnished by the appearance of the cornea, a small spot, well defined, situated in the centre is found which, by a vivid imagination, might be made to resemble a puncture produced by the ovipositor of a fly. Careful microscopic search has, however, failed to show the presence of the ovum or any other foreign body.

The symptoms of "blinds," or purulent conjunctivitis, begin as in the mild form, but the mucous secretion gives place early to pus, the conjunctiva becomes more inflamed and swollen, and in consequence of this the cornea suffers, often ulcers forming in its tissue and frequently necrosis of the entire cornea through malnutrition. The purulent secretion is contagious and many animals become infected by actual contact.

The lids are very hot and swollen and purulent matter oozes out of the lids and runs down on the face, both eyes being in tho same condition usually. Prolapse of the iris often happens and in some instances the whole eye is lost from general inflammation.

Treatment of such cases varies according to the special symptoms.

When large numbers of cattle or sheep are attacked the best thing is to remove them from an exposed situation and give them such shelter from sun and wind as may be available, isolating carefully the well from the diseased. Cleanliness is invaluable in such cases, as the secretion is very ichorous. The eyes should be washed out at least four times daily with a saline wash, or by means of a solution of boracic acid, twenty grains to the pint.

In the severe cases the upper lid should be everted and an application of nitrate of silver, ten grains to the ounce of water, made by a camel's hair pencil; this may be made twice daily. The cornea should not be touched by the silver, as suggested by so many authors on veterinary medicine, as a deposit of this salt will tend to increase the corneal opacity, thus impairing the future usefulness of the eye.

In the mild case the alum solution before mentioned may be used.

If the cornea has a deep ulcerated surface a solution of sulphate of atropia, grains two, to water one ounce will be found useful in dilating the pupil and also in allaying the pain. This solution may be used three times a day, using two or three drops at a time.

When the bowels are inactive a good dose of Epsom salts will be found a useful adjunct to the other measures.

Often in "blinds" the nasal mucous membrane is highly inflamed, in fact the eye trouble often arises in that way. It is well to syringe the nose with a warm solution of boracic acid that the secretion may be removed and the germs destroyed by the anti germicide.

After the disease has passed away the cornea presents many varied aspects, opacities, more or less extensive, according as the disease has been pronounced. The eye-lids often need treatment after the purulent inflammation has subsided, as the conjunctiva may become granular, keeping up thus a weak and watery eye. This granular condition may be treated by sulphate of copper in crystal, applied to the inner surface of the lids.

133 East 38th Street, N. Y. City.

*(To be continued.)*

